# Biologic Therapy of Leukemia

**DOI:** 10.1038/sj.bjc.6601432

**Published:** 2004-01-06

**Authors:** J Chang

**Affiliations:** 1 Christie Hospital NHS Trust, Manchester, UK

This short book of 269 pages contains a series of concise chapters, that are clearly grouped into topics. All the chapters have a uniformity of presentation. The literature is extensively reviewed; the science and the therapeutic applications are well explained and discussed. Inevitably, some repetition occurs. This book successfully presents scientific data up to 2002 and is a useful and comprehensive reference book for both clinicians and scientists.

The topics covered are:

1. Immunotherapy
2. The use of cytokines
3. Targeted strategies, eg, antisense therapy, signal transduction inhibitors, etc.
4. Differentiation agents
5. Gene therapy

Clinicians who treat leukaemia will find this book very useful for up-to-date information and references, as recent and experimental treatment modalities are well discussed. The rationales for the treatment schedules are presented clearly, so the clinicians can judge the usefulness of these regimens.

The leukaemias provide excellent material for studies on the mechanisms of cell proliferation and inhibition, receptors and signalling pathways and the data contained in the book are also very relevant to cancer physicians. Most of the science and experimental data presented relate to Oncology in general.

Overall, this book is highly recommended for its up-to-date discussion and review of the science behind various forms of treatment for leukaemia.

